# SOV_refine: A further refined definition of segment overlap score and its significance for protein structure similarity

**DOI:** 10.1186/s13029-018-0068-7

**Published:** 2018-04-20

**Authors:** Tong Liu, Zheng Wang

**Affiliations:** Department of Computer Science, University of Miami, 1365 Memorial Drive, Coral Gables, FL 33124 USA

**Keywords:** Segment overlap score, SOV score, Protein secondary structure prediction, Assessment of protein secondary structure predictions, Protein structure similarity, Similarity of segmented biological sequences, Comparing different definitions of topologically associating domains

## Abstract

**Background:**

The segment overlap score (SOV) has been used to evaluate the predicted protein secondary structures, a sequence composed of helix (H), strand (E), and coil (C), by comparing it with the native or reference secondary structures, another sequence of H, E, and C. SOV’s advantage is that it can consider the size of continuous overlapping segments and assign extra allowance to longer continuous overlapping segments instead of only judging from the percentage of overlapping individual positions as Q3 score does. However, we have found a drawback from its previous definition, that is, it cannot ensure increasing allowance assignment when more residues in a segment are further predicted accurately.

**Results:**

A new way of assigning allowance has been designed, which keeps all the advantages of the previous SOV score definitions and ensures that the amount of allowance assigned is incremental when more elements in a segment are predicted accurately. Furthermore, our improved SOV has achieved a higher correlation with the quality of protein models measured by GDT-TS score and TM-score, indicating its better abilities to evaluate tertiary structure quality at the secondary structure level. We analyzed the statistical significance of SOV scores and found the threshold values for distinguishing two protein structures (SOV_refine  > 0.19) and indicating whether two proteins are under the same CATH fold (SOV_refine > 0.94 and > 0.90 for three- and eight-state secondary structures respectively). We provided another two example applications, which are when used as a machine learning feature for protein model quality assessment and comparing different definitions of topologically associating domains. We proved that our newly defined SOV score resulted in better performance.

**Conclusions:**

The SOV score can be widely used in bioinformatics research and other fields that need to compare two sequences of letters in which continuous segments have important meanings. We also generalized the previous SOV definitions so that it can work for sequences composed of more than three states (e.g., it can work for the eight-state definition of protein secondary structures). A standalone software package has been implemented in Perl with source code released. The software can be downloaded from http://dna.cs.miami.edu/SOV/.

## Background

Protein secondary structure (SS) in three (H for helix, E for strand, and C for coil) or eight states as defined in [1] is a typical example of segmented sequences in bioinformatics. Besides protein secondary structure, new bioinformatics problems arose recently that were also dealing with segmented sequences. For example, topologically associating domains (TADs) were recently identified as megabase-sized self-interaction regions in mammalian genomes [2]. Given a genomic region containing several TADs, we can label the bodies of TADs as “D” and the boundary regions as “B”, resulting in a segmented sequence in two states (i.e., D and B). These cases about segmented sequences raise an issue about how to benchmark the predicted sequence against the reference one (e.g., the observed secondary structures), because evaluation methods based on individual positions, such as the Q3 score (that is equal to the ratio between the count of identical positions and the length of sequence), cannot take the length of continuous segments into consideration. Therefore, a measurement that can address this issue is in demand.

Segment overlap measure (SOV) was originally defined in [3] by Rost et al. to evaluate the quality of predicted protein secondary structures on a segment base. It takes several factors into consideration including the number of segments in a secondary structure, the averaged segment length, and the distribution of the length values. As a result, it allows some variations at the boundary regions of the segments by assigning some allowance (bonus), and can handle extreme cases (e.g., penalizing wrong predictions) reasonably by providing a sliding scale of segment overlap. However, that measure did not normalize the SOV scores into a fixed range, which makes it difficult to compare with other scores in percentage terms or in the range of 0 to 1. This problem was addressed by Zemla et al. in [4]. The modified definition of SOV (SOV’99) uses the length of all segments in the reference sequence to normalize the SOV scores for each state, which can make the final SOV score in percentage scale. Both measures define allowance (bonus) mechanisms for allowing some variations at the boundaries of segments, which are very important and can directly affect the scale of SOV scores for each state. For both of these two SOV definitions, the allowance assigned to each overlapping segment pair is determined by several factors including segment length and overlapping level and is an integer that cannot lead to a more than perfect value of SOV score (i.e., larger or equal to 1 for range 0–1). This 1999 version of SOV score (SOV’99) has been widely used as a standard measure for evaluating protein secondary structure predictions [5–14]. Currently, it also has been widely used in quality assessment (QA) of protein models as a machine learning feature [15–17].

However, our analysis will later show that the definition of allowance in SOV’99 has a significant drawback, that is, it cannot ensure the allowance being incremental when the prediction becomes better. For example, suppose there have been five continuous overlapping correctly-predicted positions (identical between predicted and reference sequence, for example, “HHHHH”), if one more position is accurately predicted, i.e., making it a six-element overlapping segment, more allowance should be given than the previous five-position case. The intuition is that accurately predicting one more position on top of a five-element segment (e.g., from “HHHHH” to “HHHHHH”) is more difficult and deserves more bonus points. However, the definition of SOV’99 cannot ensure this intuition. In this research, we further modified SOV’99 by designing a new definition of allowance and named it SOV_refine.

## Results

In this section, we first explain the advantage of SOV_refine modified from the definition of SOV’99. Specifically, we use an example to illustrate incremental allowance when one more residue is predicted accurately while keeping other advantages of SOV’99. After that, we show that SOV_refine can better indicate the three-dimensional quality of protein models at the secondary structure level. We then provide in-depth analysis of statistical significance of Q3 and SOV scores. Finally, we demonstrate two application examples of SOV scores: (1) SOV scores as machine learning features for developing quality assessment tools; (2) Evaluating the similarity of the inferred locations of TADs in mammalian genomes.

### Incremental allowance for better predictions

In order to make a direct comparison with SOV’99, here we use the same examples provided in the publication of SOV’99 [4]. As shown in Table 1, “predicted 1” seems to be a bad prediction because it does not have an H-state segment with a length larger than two. Therefore, although it has a relatively high Q3 value, the two SOV scores (SOV’99 and our SOV_refine) are relatively small (both punish this case to different degrees compared to Q3 score). It can be found that the SOV_refine score is slightly larger than the SOV’99 score in this case. This is because the amount of allowance assigned by SOV’99 in this case is zero [4], whereas our SOV_refine is designed to assign a larger-than-zero allowance, in this case to the H-state segment. Obviously, SOV’99 gives this case a larger punishment than our method SOV_refine (SOV’99 gives a zero allowance and a lower overall score than SOV_refine does). However, it should be noticed that although “predicted_1” is a bad prediction, it is not completely wrong because it does accurately predict some isolated states in a couple of positions (it’s just that the accurately predicted positions are not adjacent to each other). However, SOV’99 assigns a harsh punishment by assigning allowance zero, but our SOV_refine still assigns a small allowance, small enough to show that it is a bad prediction that deserves a SOV_refine score to be much lower than Q3 score (i.e. some punishments compared to Q3 score), but meanwhile not as low as a zero allowance, to indicate “predicted 1” is not completely wrong. This makes our SOV_refine more reasonable because it not only can punish bad predictions compared to Q3 score (our SOV_refine gives a much lower score than Q3 score for this example) but also does not give an extremely low allowance (zero) for the bad predictions such as “predicted 1” as it does accurately predict some isolated states.

**Table 1:** Examples of assessment of secondary structure predictions using Q3, SOV’99, and SOV_refine (λ = 1)

Index	Sequence	Q3	SOV’99	SOV_refine (λ = 1)
Reference	**CHHHHHHHHHHC**	–	–	–
Predicted 1	**CHCHCHCHCHCC**	0.583	0.125	0.149
Predicted 2	**CHHHCHHHCHHC**	0.833	0.406	0.371
Predicted 3	**CHHCCHHHHHCC**	0.75	0.523	0.464
Predicted 4	**CCCHHHHCCCCC**	0.50	0.544	0.459
Predicted 5	**CCCHHHHHCCCC**	0.583	0.632	0.567
Predicted 6	**CCCHHHHHHCCC**	0.667	0.806	0.678
Predicted 7	**CCCHHHHHHHCC**	0.75	0.903	0.797
Predicted 8	**CCCHHHHHHHHC**	0.833	0.944	0.937

Notice that predicted 3 and 4 indicate another different feature between SOV’99 and SOV_refine. Predicted 3 correctly predicts seven helices (in two segments) while Predicted 4 correctly predicts four helices (in one segment). In this situation, SOV_refine assigns a higher score to predicted 3 as seven correct helices are more consistent to the reference’s 10 helices compared to predicted 4’s four helices. However, SOV’99 in this case assigns a higher score to predicted 4 showing it prefers one segment prediction even though the number of accurately predicted residues is largely different

The next two predictions (i.e., “predicted 2” and “predicted 3”) have longer H-state segments, resulting in larger SOV scores. Predictions 4 through 8 are deliberately selected to demonstrate the essential difference between SOV’99 and our SOV_refine when one more element (i.e., H-state residue in predicted assignments) is further predicted accurately. As expected, the accuracy for Q3 is increased by a fixed value of 0.083. For SOV’99, the scores are irregularly increased by 0.008, 0.174, 0.097, and 0.041, while the scores from SOV_refine are increased by 0.108, 0.111, 0.119, and 0.14, which keep increasing when the predictions are getting better.

The two different observations can be properly explained from the distinct definitions of assigning allowance from SOV’99 and SOV_refine. To be specific, SOV’99 cannot ensure the amount of allowance stably increased, whereas SOV_refine is designed to be capable of handling this case.

We provide another example with a reference sequence composed of four states shown in Table 2 to demonstrate that SOV_refine can assign distinguishable scores by adjusting λ parameter in the definition. There are four predictions, which are getting better from predicted 1 up to predicted 4. SOV’99 and SOV_refine (λ = 1) cannot distinguish which one is better between predicted 3 and predicted 4, whereas SOV_refine with λ equal to 0.5 or equal to 0.1 can conclude that predicted 4 is better than predicted 3. This example indicates that the definition of SOV_refine is more flexible than that of SOV’99. It can be found that a smaller λ will make the SOV_refine algorithm more stringent. Table 2 lists all the SOV_refine scores with different λ values, from which users can pick up the appropriate λ value based on their stringency demands of their specific problems. In our implementation, the default value of λ is 1.

**Table 2:** A reference sequence with four states (i.e., A, B, C, and D) compared with four predicted sequences using Q4 (Accuracy), SOV’99, and SOV_refine with different λ values

Index	Sequence	Q4	SOV’99	SOV_refine		
				λ = 1	λ = 0.5	λ = 0.1
Reference	**AABBBBBBCCCCCCDD**	–	–	–	–	–
Predicted 1	**AAAAABBBCCCDDDDD**	0.625	0.65	0.807	0.641	0.508
Predicted 2	**AAAABBBBCCCCDDDD**	0.75	0.938	0.925	0.85	0.67
Predicted 3	**AAABBBBBCCCCCDDD**	0.875	1.0	1.0	0.961	0.851
Predicted 4	**AABBBBBBCCCCCDDD**	0.938	1.0	1.0	0.981	0.925

### Evaluation of protein tertiary models at the secondary structure level

We downloaded the protein native structures and predicted models of 33 Template-Based Modeling (TBM) single-domain targets in the Critical Assessment of protein Structure Prediction 11 (CASP11) at http://www.predictioncenter.org/casp11/. The native structures for the 33 single-domain targets are available at CASP official website. For each target, 20 protein models in stage 1 for quality assessment (QA) are chosen as the predicted structures [18], because these models cover the whole range of model accuracy.

We then superimposed the 20 models of each target with their native structure using three different protein structure alignment tools: LGA [19], TM-align [20], and DeepAlign [21], resulting in a set of scores (i.e., GDT-TS from LGA, TM-score from TM-align, GDT-TS from DeepAlign, and TM-score from DeepAlign) for measuring the quality of predicted protein 3D models from three-dimensional superimposing. After that, secondary structures of 33 native structures and their corresponding models were assigned by STRIDE [22] and DSSP [1] in three states (i.e., H, E, and C), respectively. Overall, we obtained four sets of 660 GDT-TS or TM-score and 660 pairs of observed and predicted secondary structures, for each pair of which we carried out the comparisons of secondary structures using three measures: Q3 score for three-state secondary structure, SOV’99, and SOV_refine (λ = 1), respectively.

**Fig. 1:**
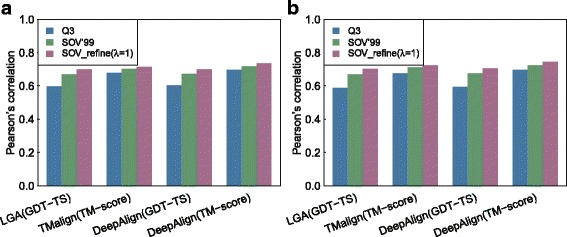
Assessment of predicted protein tertiary structures at the secondary structure level. The Pearson’s correlation coefficients between 3D-based scores (GDT-TS and TM-score) for measuring the quality of predicted tertiary structures and 2D-based scores (Q3, SOV’99, and SOV_refine) for assessing the quality of predicted secondary structures: **a** using STRIDE to assign secondary structures; **b** using DSSP to assign secondary structures

**Fig. 2:**
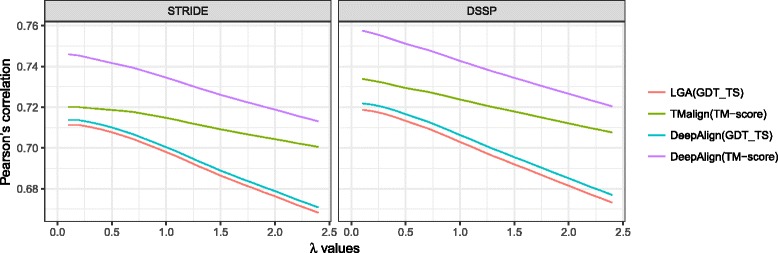
The Pearson’s correlation coefficients between GDT_TS (a 3D-based score) and SOV_refine (a 2D-based score), and between TM-score (a 3D-based score) and SOV_refine with different λ values for measuring the quality of predicted tertiary structures: **a** using STRIDE to assign secondary structures; **b** using DSSP to assign secondary structures

We then explored whether our SOV_refine can better indicate the three-dimensional quality of protein models by comparisons at the secondary structure level. The Pearson’s correlation coefficients were calculated between each of the four sets of the three-dimensional superimposing scores (GDT-TS from LGA and DeepAlign, and TM-score from TM-align and DeepAlign) and the scores of comparing secondary structures using Q3 score, SOV’99, and SOV_refine (see Fig. 1(a) for STRIDE and 1(b) for DSSP): for using STRIDE, SOV_refine (λ = 1) constantly achieves the best performance with *r* = 0.70, 0.71, 0.70, and 0.73 (*p*-value < 10^− 5^), followed by SOV’99 (*r* = 0.67, 0.70, 0.67, and 0.72), and Q3 (*r* = 0.60, 0.68, 0.60, and 0.70); for using DSSP, we can draw the same conclusion. Since the λ parameter in the definition of SOV_refine in Eq. 4 is adjustable, we have tried to check whether different λ values affect the Pearson’s correlation performance. We have tested the λ values in the range of [0.1, 2.4]. The results shown in Fig. 2 indicate that smaller λ values achieve larger Pearson’s correlation coefficients.

### Statistical significance of Q3 and SOV_refine scores

Here we aim to address two questions as described in [23]: (1) What is the statistical significance of Q3, SOV’99, and SOV_refine? (2) For a given score what is the probability of two proteins having the same fold?

**Fig. 3:**
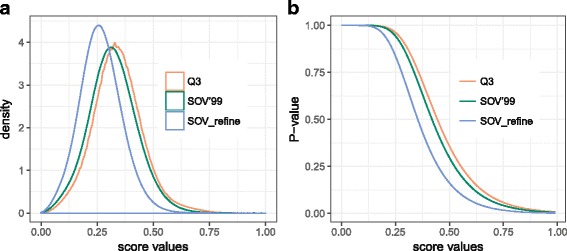
**a** The distributions of Q3, SOV’99, and SOV_refine from a sample of 14,252,776 scores calculated from Top8000 database. **b** The probabilities (*P*-values) of having a given score of Q3, SOV’99, and SOV_refine greater than or equal to a certain value. We can find that the probabilities of finding Q3 ≤ 0.26, SOV’99 ≤ 0.24, and SOV_refine ≤ 0.19 for two random proteins are close to 1. These findings indicate that we can distinguish two protein structures or models at the secondary structure level if their Q3 or SOV scores are greater than or equal to these corresponding thresholds

For the statistical significance of Q3, SOV’99, and SOV_refine, we used Top8000 database [24] including 8000 high-resolution quality-filtered protein chains. After filtering out chains with length larger than 200 or less than 80, we obtained 3420 protein chains, resulting in 5,846,490 protein pairs. For each protein pair, we calculated its Q3, SOV’99, and SOV_refine scores. If two protein chains do not have the same length, the scores were calculated between the smaller chain and a sliding window with length equal to the length of the smaller chain on the larger chain (20-residue sliding interval). We finally obtained 14,252,776 scores for Q3, SOV’99, and SOV_refine; and their distribution can be found in Fig. 3(a). The *P*-values (i.e., the probability of having a Q3, SOV’99, or SOV_refine score equal to or larger than a certain value) for a given score were calculated by the same way as in [23] and shown in Fig. 3(b). In general, when Q3 ≤ 0.26, SOV’99 ≤ 0.24, and SOV_refine ≤ 0.19, the probability of finding these scores from our sample is close to 1 (these values were found when the P-values start to decrease from 0.95), and then the P-values decrease rapidly when the scores are getting larger than these thresholds. These findings mean that in order for Q3, SOV’99, and SOV_refine to distinguish two structures from the secondary structure level, their scores need to be higher than these thresholds.

**Fig. 4:**
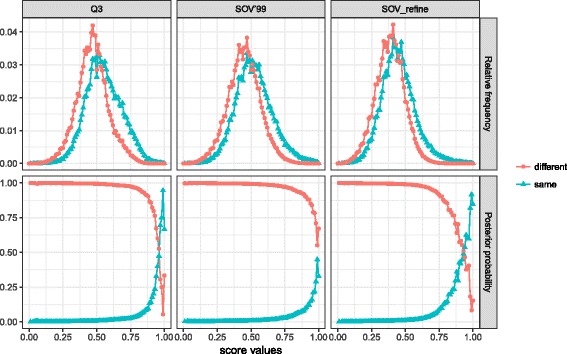
Top three plots: the relative frequency (i.e., conditional probabilities) of Q3, SOV’99, and SOV_refine for *three-state* secondary structure for protein pairs from Top8000 having the same and different CATH folds. For example, red lines indicate conditional probability $P\left({SOV}_{-} refine\left|\overline{F}\right.\right)$ of SOV_refine score when the two proteins are in different CATH fold families whereas the blue lines indicate *P*(*SOV*_−_*refine*|*F*), which is the conditional probability of SOV_refine when the two proteins are in the same CATH fold family. Bottom three plots: posterior probability of proteins with a given score of Q3, SOV’99, and SOV_refine for *three-state* secondary structure when two proteins are in the same and different CATH folds. For example, the posterior probability of two proteins to have the same fold given a specific SOV_refine score is represented by *P*(*F*|*SOV*_−_*refine*), whereas $P\left(\left.\overline{F}\right|{SOV}_{-} refine\right)$ for not having the same fold. Red lines indicate not having the same fold; and blue lines indicate having the same fold. The point when the two lines with different colors intersect is the score threshold, above which we think two proteins are having the same fold

**Fig. 5:**
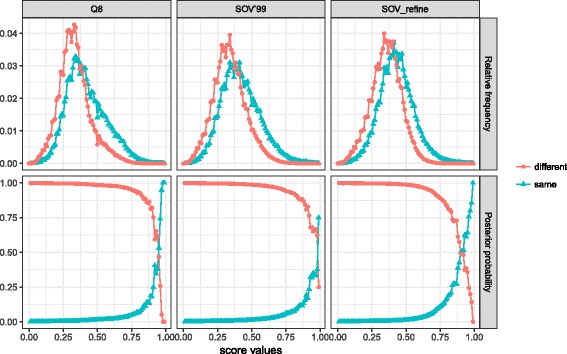
Top three plots: the relative frequency (i.e., conditional probabilities) of Q8, SOV’99, and SOV_refine for *eight-state* secondary structure for protein pairs from Top8000 having the same and different folds as defined by CATH. Bottom three plots: posterior probability for a given score of Q8, SOV’99, and SOV_refine for *eight-state* secondary structure when two proteins are in the same and different folds as defined by CATH. More explanations about the meaning of the plots can be found in the caption of Fig. 4

For the probability of protein pairs having the same fold for a given score, we downloaded the latest CATH database (v4.2) [25]. After filtering out the proteins with length less than 80 or larger than 200, we obtained 18,653 proteins, which were classified into 343 folds. For folds with size larger than 100 we only kept the first 100 proteins. We then used DSSP [1] to assign secondary structures for each protein in three and eight states. For protein pairs with different lengths, we calculated their Q3, SOV’99, and SOV_refine scores as follows: (1) The secondary structure sequence in three and eight states of the smaller protein slides gaplessly (i.e., one-residue sliding interval) along the bigger protein; (2) The final Q3 and SOV scores of the protein pair are the corresponding maximum values on all the possible sliding positions generated from the first step. For three and eight states, we generated two samples individually (two samples for three states and two samples for eight states), one including scores from the protein pairs in the same folds, and the other including scores from the protein pairs in different folds. The distributions of these scores in three and eight states are shown in Figs. 4 and 5, respectively.

The top three plots in both Figs. 4 and 5 indicate the conditional probability, for example, the red lines indicate *P*(*SOV*_*refine*| *F*), which is the conditional probability of SOV_refine score when the two proteins are in different CATH fold families whereas the blue lines indicate $P\left({SOV}_{-} refine\left|\overline{F}\right.\right)$, which is the conditional probability of SOV_refine when the two proteins are in the same CATH fold family.

For a given score of Q3, SOV’99, and SOV_refine, we also calculated its posterior probabilities (bottom three plots in Figs. 4 and 5) that the two proteins have the same or different fold as the way described in [23]. For example, the posterior probability of two proteins to have the same fold given a specific SOV_refine score is represented by *P*(*F*|*SOV*_−_*refine*), whereas $P\left(\left.\overline{F}\right|{SOV}_{-} refine\right)$ for not having the same fold. The results are shown in Fig. 4 for three-state and Fig. 5 for eight-state secondary structures with red lines indicating not having the same fold and blue lines indicating having the same fold. The point when the two lines with different colors intersect is the score threshold, above which we think two proteins are having the same fold.

From Figs. 4 and 5, we can observe that scores in the same folds are slightly larger than those in different folds, but not as noticeable as the observation obtained by using TM-score as in [23]. We can also conclude that for three states when a given score from two random proteins meets Q3 ≤ 0.97 and SOV_refine ≤ 0.94, the two proteins have high probability sharing different folds; for eight states when a given score from two random proteins meets Q8 ≤ 0.95 and SOV_refine ≤ 0.90, the two proteins have high probability sharing different folds. This type of conclusion cannot be drawn for SOV’99 based on the data indicating another advantage of our SOV_refine compared to SOV’99.

### Application of SOV_refine for protein quality assessment

We proved that SOV scores, especially SOV_refine, are effective machine learning features for protein quality assessment. We used 85 targets from CASP9 and 67 targets from CASP10 as training data and their real GDT_TS scores as objective values. For each target, we randomly selected 150 protein models. For each model, we extracted 32 features, mostly from [17], as the basic feature set and generated three more feature sets: (1) Basic set plus SOV’99 for predicted and assigned secondary structures; (2) Basic set plus SOV_refine (λ = 0.1) for predicted and assigned secondary structures; (3) Basic set plus SOV_refine (λ = 1) for predicted and assigned secondary structures. We used SCRATCH [26] to obtain the predicted secondary structures. We used Random Forest [27] to train the prediction models.

We blindly tested the performance of the QA models trained from the four feature sets on 75 targets in CASP11 in two stages [18]. The evaluation measures are the same as those in official CASP evaluations [18] including (1) the weighted mean of Pearson’s product moment correlation coefficient (wmPMCC), (2) the average loss (Ave loss), (3) the average GDT_TS deviations (Ave ΔGDT), and (4) the Matthews correlation coefficient (MCC). The blind test results are shown in Table 3. All of the three SOV scores play a positive role in improving the performance; and SOV_refine (λ = 1) performs better than SOV_refine (λ = 0.1) and SOV’99 in terms of most of the evaluation criteria.

**Table 3:** The evaluation results of quality assessment of protein models using different SOV scores as machine learning features

	Stage 1			Stage 2			MCC
	wmPMCC	Ave loss	Ave ΔGDT	wmPMCC	Ave loss	Ave ΔGDT	
Basic	0.70	0.102	0.00381	0.39	0.068	0.00083	0.736
Basic + SOV’99	0.71	0.093	0.00368	0.42	0.074	0.00083	0.733
Basic + SOV_refine (λ = 0.1)	0.71	0.096	0.00368	0.41	0.073	0.00083	0.740
Basic + SOV_refine (λ = 1)	0.71	0.084	0.00361	0.40	0.066	0.00084	0.750

### SOV_refine scores for measuring similarity of different definitions of topologically associating domains (TADs)

The SOV scores have other important applications in comparing segmented sequences besides protein secondary structure sequences. Here we demonstrate an example of using SOV score to measure the similarity of different definitions (in terms of genomic locations) of topologically associating domains (TADs) in mammalian genomes. For the same genomic region, different TAD-detection algorithms may infer different TAD locations [28]. Therefore, SOV score can be used here to measure the similarity of different TAD definitions (i.e., the similarity about which part of the genomic region is within a TAD body and which part is within the boundary).

**Fig. 6:**
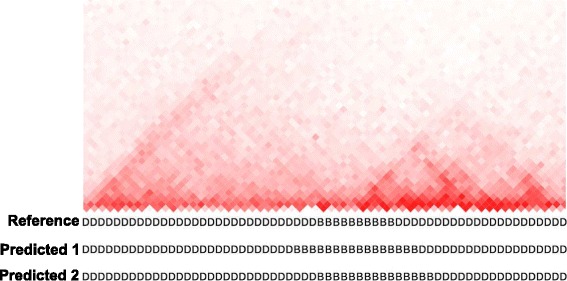
The two-dimensional heat map of normalized Hi-C interaction counts in a genomic region (Chr. 2: 137.8 Mb – 140.28 Mb) with the reference TAD definition followed by two different inferred TAD definitions (i.e., predicted 1 and predicted 2)

The normalized Hi-C data for male mouse embryonic stem cells (mESC) was downloaded from Ren Lab's website at http://chromosome.sdsc.edu/mouse/hi-c/download.html. As shown in Fig. 6, we selected a genomic region (137.8 Mb – 140.28 Mb) on chromosome 2. There are two TADs in this region based on the definition from Ren Lab http://chromosome.sdsc.edu/mouse/hi-c/download.html. However, the boundary regions between the two TADs are vague as the Hi-C signals are not sharply distinguishable. Therefore, different TAD-detection algorithms may give different definitions about the locations of the two TADs (or the boundary regions). To illustrate this, we artificially made up the reference definition (based on Ren Lab’s definition) and two other definitions as inference definitions. We labeled “D” for the positions within a TAD body and “B” for positions within a boundary region. In this way, we have three sequences (i.e., one reference and two inferences), each containing two states. The SOV’99 and SOV_refine (λ = 1) between reference and inference 1 (we use “predicted 1” in the figure to match previous examples) are 0.99 and 0.91 respectively. The SOV’99 and SOV_refine (λ = 1) between reference and predicted 2 are 1.0 and 0.89 respectively. It can be found that SOV’99 scores indicate that the two predictions are almost the same as the reference, which is actually not. However, SOV_refine scores can quantitatively detect the differences by giving a lower score, demonstrating another advantage of SOV_refine compared to SOV’99.

## Discussion

One may argue that the SOV score which was originally introduced in 1990s and protein secondary structure prediction have already been an old topic and achieved stable performance. Therefore, SOV score may not be needed. However, we found that the SOV score was still being used as a machine learning feature, for example, for protein model quality assessment. Moreover, we tested SOV’99, our SOV_refine, and Q3 on 660 TBM single-domain protein models and found that SOV_refine can indicate the three-dimensional quality (indicated by GDT-TS and TM-score) of protein models by comparisons at the secondary structure level. Furthermore, we demonstrated SOV score’s usefulness in a newly-emerged bioinformatics problem of inferring TAD locations in mammalian genomes, showing SOV score still could be widely used in bioinformatics research.

## Conclusions

In this article, we presented a further modified definition of segment overlap measures (SOV_refine) based on the definition released in 1999 (SOV’99). Specifically, we redefined the assignment of allowance for the overlapping segment pairs. The original definition of allowance has obvious drawbacks and is only based on the overlap level and length of segments. Here we provided a new definition according to the prediction difficulty of reference sequence and the local performance of predicted segments. It can ensure that the amount of allowance is increased when more elements in the segment of a predicted sequence are further predicted accurately.

We performed analysis on the statistical significance of Q3, SOV’99, and SOV_refine and concluded that the probability of finding Q3 ≤ 0.26, SOV’99 ≤ 0.24, and SOV_refine ≤ 0.19 for two random proteins was close to 1. These findings indicate that we can distinguish two protein structures or models at the secondary structure level if their Q3 or SOV scores are greater than or equal to these corresponding thresholds. We can also conclude that for three-state secondary structure when a given score from two random proteins meets Q3 ≤ 0.97 and SOV_refine ≤ 0.94, the two proteins have high probability sharing different CATH folds; for eight-state secondary structure when a given score from two random proteins meets Q8 ≤ 0.95 and SOV_refine ≤ 0.90, the two proteins have high probability sharing different CATH folds. These results also indicate that compared to TM_score we need to get a higher Q3 or SOV_refine scores of any two protein structures or models to determine whether they share the same fold. Meanwhile, we also observed that the two-dimensional alignment scores (i.e., Q3, SOV’99, and SOV_refine) are not as effective as the three-dimensional alignment scores (i.e., TM-score) when they are used to determine whether two random proteins have the same fold.

We provided another two applications to demonstrate the advantages of SOV_refine compared to SOV’99. One of them is to use SOV scores as features in machine-learning tools for quality assessment of protein models. Our evaluation results show that adding SOV_refine into the basic machine learning feature set results in a larger improvement on performance compared to adding SOV’99. The other application is to use SOV scores as similarity measure for different TAD definitions. The results show that SOV_refine can better distinguish the obvious difference in TAD definitions, whereas SOV’99 often assigns false perfect scores.

We implemented SOV_refine and re-implemented SOV’99 as a standalone computer program. Technically, it can handle unlimited number of states in a reference sequence. However, we highly recommend not to use them when the number of states is quite large (e.g., > 10 states) because more states will reduce the usefulness and significance of SOV scores, in which case the accuracy on a per-element base (e.g., Q3) would be more suitable.

## Methods

In this section, we describe the definition of SOV_refine in detail. For the purpose of consistency, we use the same denotations as used in [4]. Here, the reference *s*_*r*_ and predicted *s*_*p*_ sequences are respectively the native and predicted assignments of protein secondary structures in three states (i.e., H, E, and C); however, our program can handle unlimited number of states and optional labels for states, even though it may not make much sense if the number of states is too large.

The SOV score for each state *i*, *SOV(i)*, is calculated individually, and then the global SOV score is a weighted combination of individual state scores. Let *s*_*1*_ be a segment in state *i* in *s*_*r*_ and *s*_*2*_ in state *i* in *s*_*p*_. A pair of overlapping segments is denoted as (*s*_*1*_, *s*_*2*_); and the set of these pairs for state *i* is *S*(*i*) = {(*s*_1_,  *s*_2_)| *s*_1_ ⋂ *s*_2_ ≠ ∅}. If given *s*_*1*_, there are no overlapping segments *s*_*2*_, then we define another set *S*^′^(*i*) = {(*s*_1_,   ∀ *s*_2_)| *s*_1_ ⋂ *s*_2_ = ∅}. The *SOV(i)* is defined as follows:

1 $$ SOV(i)=\frac{1}{N(i)}\times \sum \limits_{S(i)}\left[\frac{\min ov\left({s}_1,{s}_2\right)+\delta \left({s}_1,{s}_2\right)}{\max ov\left({s}_1,{s}_2\right)}\times len\left({s}_1\right)\right] $$

where *len(s*_*1*_*)* is the number of elements in segment *s*_*1*_; *minov(s*_*1*_*, s*_*2*_*)* is the number of identical (actually overlapping in *i*-state) elements in both *s*_*1*_ and *s*_*2*_, while *maxov(s*_*1*_*, s*_*2*_*)* is the total number of elements for which either of the two segments is assigned state *i*; δ(s_1_, s_2_) is the amount of allowance assigned to the pair. *N(i)* is the normalization value defined as:

2 $$ N(i)=\sum \limits_{S(i)} len\left({s}_1\right)+\sum \limits_{S^{\prime }(i)} len\left({s}_1\right) $$

The new definition of allowance is:

3 $$ \delta \left({s}_1,{s}_2\right)=\delta (all)\times \frac{len\left({s}_1\right)}{len\left({s}_r\right)}\times \frac{\min ov\left({s}_1,{s}_2\right)}{\max ov\left({s}_1,{s}_2\right)} $$

where *len(s*_*r*_*)* is the number of elements in *s*_*r*_; and *δ(all)* is the total allowance assigned to the whole reference sequence: it can be a fixed value for all reference sequences or depends on each individual sequence. For example, if *δ(all)* = 1, then all allowance values for segment pairs should be less than or equal to one. Considering that it is difficult to determine a proper fixed value of *δ(all)*, we further define it using the number of states *N*_*C*_ and the length of all segments in *s*_*r*_:

4 $$ \delta (all)=\lambda \times \frac{N_C}{\sum_{j=1}^{N_S}{\left(\frac{len\left({s}_j\right)}{len\left({s}_r\right)}\right)}^2} $$

where *N*_*S*_ is the number of segments in *s*_*r*_; *s*_*j*_ is the *j-*th segment; λ is an adjustable scale parameter and used to limit the range of *δ(all)*. In the test example for assessing the quality of predicted protein secondary structures, λ equaling to 1 is acceptable. Eq. 4 is designed based on two intuitive facts: (1) More allowance should be assigned when the number of states in *s*_*r*_ is larger because it makes the prediction difficult; (2) More allowance should be assigned when the weighted average length (the denominator part in Eq. 4) of all segments in *s*_*r*_ is smaller because a small average length results in more boundary regions, which increases the difficulty of predictions. In order to avoid more than perfect for *SOV(i)*, when the amount of allowance calculated in Eq. 3 is larger than (*maxov(s*_*1*_*,s*_*2*_*)-minov(s*_*1*_*,s*_*2*_*)*) the allowance is set to (*maxov(s*_*1*_*,s*_*2*_*)-minov(s*_*1*_*,s*_*2*_*)*).

Suppose that the number of states in *s*_*r*_ is *N*_*C*_, then the final SOV score, *SOV_refine*, can be defined as:

5 $$ SOV\_ refine=\frac{\sum_{i=1}^{N_C}\left( SOV(i)\times N(i)\right)}{\sum_{i=1}^{N_C}N(i)} $$

The new definition of SOV_refine remedies three deficiencies found in SOV’99. First, the amount of allowance does not have to be an integer. Instead, the amount of allowance defined in Eq. 3 is based on the local performance of *s*_*2*_ and a fractional part of *δ(all)*. Second, SOV’99 cannot ensure that the amount of allowance keeps increasing when more residues in a segment in *s*_*p*_ are further predicted accurately, whereas SOV_refine can. Third, we take the allowance for the whole reference sequence *s*_*r*_ into consideration, because sometimes it may be much easier to predict (e.g., when *s*_*r*_ only has one state), while for other cases it may be very difficult (e.g., if *s*_*r*_ has eight states and multiple segments with different lengths). In our design, the value of *δ(all)* depends on *s*_*r*_, that is, reference sequences with different lengths and prediction difficulty have different *δ(all)*.

## Abbreviations

3D: Three-dimensional
Q3: Three states for protein secondary structure
QA: Quality assessment
SOV: Segment overlap measures
TAD: Topologically Associating Domain
TBM: Template-Based Modelling

## References

[1] Kabsch W, Sander C (1983). Dictionary of protein secondary structure: pattern recognition of hydrogen-bonded and geometrical features. Biopolymers.

[2] Dixon JR, Selvaraj S, Yue F, Kim A, Li Y, Shen Y, Hu M, Liu JS, Ren B (2012). Topological domains in mammalian genomes identified by analysis of chromatin interactions. Nature.

[3] Rost B, Sander C, Schneider R (1994). Redefining the goals of protein secondary structure prediction. J Mol Biol.

[4] Zemla A, Venclovas Č, Fidelis K, Rost B (1999). A modified definition of Sov, a segment-based measure for protein secondary structure prediction assessment. Proteins: Structure Function Bioinformatics.

[5] Wang S, Peng J, Ma J, Xu J (2016). Protein secondary structure prediction using deep convolutional neural fields. Sci Rep.

[6] Aloy P, Stark A, Hadley C, Russell RB (2003). Predictions without templates: new folds, secondary structure, and contacts in CASP5. Proteins: Structure Function Bioinformatics.

[7] Jones D (1999). Protein secondary structure prediction based on position-specific scoring matrices. J Mol Biol.

[8] Geourjon C, Deleage G (1995). SOPMA: significant improvements in protein secondary structure prediction by consensus prediction from multiple alignments. Comp Applicat Biosci.

[9] Kim H, Park H (2003). Protein secondary structure prediction based on an improved support vector machines approach. Protein Eng.

[10] Ward JJ, McGuffin LJ, Buxton BF, Jones DT (2003). Secondary structure prediction with support vector machines. Bioinformatics.

[11] Guermeur Y, Geourjon C, Gallinari P, Del G (1999). Improved performance in protein secondary structure prediction by inhomogeneous score combination. Bioinformatics.

[12] Pollastri G, Mclysaght A (2005). Porter: a new, accurate server for protein secondary structure prediction. Bioinformatics.

[13] Hua S, Sun Z (2001). A novel method of protein secondary structure prediction with high segment overlap measure: support vector machine approach. J Mol Biol.

[14] Martin J, Letellier G, Marin A, Taly J-F, de Brevern AG, Gibrat J-F (2005). Protein secondary structure assignment revisited: a detailed analysis of different assignment methods. BMC Struct Biol.

[15] Wang Z, Eickholt J, Cheng J (2011). APOLLO: a quality assessment Service for Single and Multiple Protein Models. Bioinformatics.

[16] Cao R, Wang Z, Wang Y, Cheng J (2014). SMOQ: a tool for predicting the absolute residue-specific quality of a single protein model with support vector machines. BMC Bioinform.

[17] Liu T, Wang Y, Eickholt J, Wang Z (2016). Benchmarking deep networks for predicting residue-specific quality of individual protein models in CASP11. Sci Rep.

[18] Kryshtafovych A, Barbato A, Monastyrskyy B, Fidelis K, Schwede T, Tramontano A. Methods of model accuracy estimation can help selecting the best models from decoy sets: assessment of model accuracy estimations in CASP11. Proteins: Structure Function Bioinformatics. 2016;84(S1):349–69. 10.1002/prot.24919 PMC4781682 26344049

[19] Zemla A (2003). LGA: a method for finding 3D similarities in protein structures. Nucleic Acids Res.

[20] Zhang Y, Skolnick J (2005). TM-align: a protein structure alignment algorithm based on the TM-score. Nucleic Acids Res.

[21] Wang S, Ma J, Peng J, Xu J (2013). Protein structure alignment beyond spatial proximity. Sci Rep.

[22] Frishman D, Argos P (1995). Knowledge-based protein secondary structure assignment. Proteins Struct Funct Genet.

[23] Xu J, Zhang Y (2010). How significant is a protein structure similarity with TM-score= 0.5?. Bioinformatics.

[24] Chen VB, Arendall WB, Headd JJ, Keedy DA, Immormino RM, Kapral GJ, Murray LW, Richardson JS, Richardson DC (2010). MolProbity: all-atom structure validation for macromolecular crystallography. Acta Crystallogr D Biol Crystallogr.

[25] Sillitoe I, Lewis TE, Cuff A, Das S, Ashford P, Dawson NL, Furnham N, Laskowski RA, Lee D, Lees JG (2014). CATH: comprehensive structural and functional annotations for genome sequences. Nucleic Acids Res.

[26] Magnan CN, Baldi P (2014). SSpro/ACCpro 5: almost perfect prediction of protein secondary structure and relative solvent accessibility using profiles, machine learning and structural similarity. Bioinformatics.

[27] Breiman L (2001). Random forests. Mach Learn.

[28] Dali R, Blanchette M (2017). A critical assessment of topologically associating domain prediction tools. Nucleic Acids Res.

